# Supplementary material for: Identification and functional analysis of non-coding regulatory small RNA FenSr3 in Bacillus amyloliquefaciens LPB-18
Source: PeerJ. 2023 May 15;11:e15236. doi: 10.7717/peerj.15236 (PMC10194069; doi:10.7717/peerj.15236)
Supplement: Supplemental Information 4 [file peerj-11-15236-s004.zip › KO/CK-vs-T1_map/map05215.html]

KEGG PATHWAY: Prostate cancer - Reference pathway


|  |  |
| --- | --- |
| **Prostate cancer - Reference pathway** |  |

[
Pathway menu
| Organism menu
| Pathway entry
| Show description
| User data mapping
]

Prostate cancer constitutes a major health problem in Western countries. It is the most frequently diagnosed cancer among men and the second leading cause of male cancer deaths. The identification of key molecular alterations in prostate-cancer cells implicates carcinogen defenses (GSTP1), growth-factor-signaling pathways (NKX3.1, PTEN, and p27), and androgens (AR) as critical determinants of the phenotype of prostate-cancer cells. Glutathione S-transferases (GSTP1) are detoxifying enzymes. Cells of prostatic intraepithelial neoplasia, devoid of GSTP1, undergo genomic damage mediated by carcinogens. NKX3.1, PTEN, and p27 regulate the growth and survival of prostate cells in the normal prostate. Inadequate levels of PTEN and NKX3.1 lead to a reduction in p27 levels and to increased proliferation and decreased apoptosis. Androgen receptor (AR) is a transcription factor that is normally activated by its androgen ligand. During androgen withdrawal therapy, the AR signal transduction pathway also could be activated by amplification of the AR gene, by AR gene mutations, or by altered activity of AR coactivators. Through these mechanisms, tumor cells lead to the emergence of androgen-independent prostate cancer.

|  |  |  |
| --- | --- | --- |
| Reference pathway | 184% 150% 122% 100% 82% 67% 55% | 图片下载 |
